# Efficacy and safety of tofacitinib in the treatment of rheumatoid arthritis: a systematic review and meta-analysis

**DOI:** 10.1186/1471-2474-14-298

**Published:** 2013-10-18

**Authors:** Ying He, Angel YS Wong, Esther W Chan, Wallis CY Lau, Kenneth KC Man, Celine SL Chui, Alan J Worsley, Ian CK Wong

**Affiliations:** 1 Centre for Safe Medication Practice and Research, Department of Pharmacology and Pharmacy, The University of Hong Kong, Pokfulam, Hong Kong

**Keywords:** Tofacitinib, Rheumatoid arthritis, Treatment outcome, Meta-analysis

## Abstract

**Background:**

Tofacitinib is a disease-modifying antirheumatic drug (DMARD) which was recently approved by US Food and Drug Administration (FDA). There are several randomised clinical trials (RCTs) that have investigated the efficacy and safety of tofacitinib in adult patients with rheumatoid arthritis (RA). A systematic review with a meta-analysis of RCTs was undertaken to determine the efficacy and safety of tofacitinib in treating patients with RA.

**Methods:**

Electronic and clinical trials register databases were searched for published RCTs of tofacitinib between 2009 and 2013. Outcomes of interest include 20% and 50% improvement in the American College of Rheumatology Scale (ACR20 and ACR50) response rates, rates of infection, the number of immunological/haematological adverse events (AEs), deranged laboratory results (hepatic, renal, haematological tests and lipoprotein level) and the incidence of drug withdrawal.

**Results:**

Eight RCTs (n = 3,791) were reviewed. Significantly greater ACR20 response rates were observed in patients receiving tofacitinib 5 and 10 mg bid (twice daily) versus placebo at week 12, with risk ratios (RR) of 2.20 (95% CI 1.58, 3.07) and 2.38 (95% CI 1.81, 3.14) respectively. The effect was maintained at week 24 for 5 mg bid (RR 1.94; 95% CI 1.55, 2.44) and 10 mg bid (RR 2.20; 95% CI 1.76, 2.75). The ACR50 response rate was also significantly higher for patients receiving tofacitinib 5 mg bid (RR 2.91; 95% CI 2.03, 4.16) and 10 mg bid (RR 3.32; 95% CI 2.33, 4.72) compared to placebo at week 12. Patients in the tofacitinib group had significantly lower mean neutrophil counts, higher serum creatinine, higher percentage change of LDL/HDL and a higher risk of ALT/AST > 1 ULN (upper limit of normal) versus placebo. There were no significant differences in AEs and withdrawal due to AEs compared to placebo.

**Conclusion:**

Tofacitinib is efficacious and well tolerated in patients with MTX-resistant RA up to a period of 24 weeks. However, haematological, liver function tests and lipoproteins should be monitored. Long-term efficacy and pharmacovigilance studies are recommended.

## Background

Rheumatoid arthritis (RA) is a chronic inflammatory arthritis and a systemic autoimmune disease, which can lead to long-term joint damage, loss of function and disability [1]. In addition to the symptoms of joint destruction such as pain, swelling, or stiffness of the joints, those patients with RA may also suffer from other extra-articular manifestations, namely rheumatoid nodules [2], interstitial lung disease [3], cardiac involvement [4], and Felty’s syndrome [5]. The onset of RA typically occurs between 30 to 50 years age. In the United Kingdom, the highest incidence is observed in people over 70 years of age [6]. It is estimated that the adult prevalence of RA is 0.5-1% in Europe and 1% in the United States [1,7,8]. Within two years of onset, approximately one third of people with RA are unable to continue with employment due to the disease [6]. The mortality rate of those people with RA is approximately twice of those without [9].

Guidelines for the management of RA have been issued by the American College of Rheumatology (ACR) [7,10], and by the National Institute for Health and Clinical Excellence guidelines in 2009 (NICE CG79) [6]. It is suggested that there are three main steps in the management of RA: pharmacological, non-pharmacological and surgical treatment [6,7].

Pharmacological treatment of RA generally includes: non-steroidal anti-inflammatory drugs (NSAIDs), glucocorticoid treatment, disease-modifying antirheumatic drugs (DMARDs), and biologic agents [7,10]. By treating inflammation, relieving pain and/or suppressing the immune response, NSAIDs and glucocorticoids are able to alleviate some of the symptoms associated with RA. Currently, DMARDs such as methotrexate (MTX) remain the main treatment approach [7]. Antirheumatic biologics, including the tumor necrosis factor (TNF) inhibitors such as adalimumab or non-TNF inhibitors [10] are usually considered when the other treatment approaches are not sufficiently effective. There are still about one third of patients who have an unsatisfactory response to available treatments [11]. Consequently, development of new drugs and therapy for RA is needed.

Tofacitinib (CP-690550), also called tasocitinib [12] during early development with the commercial name Xeljanz®, is a new oral DMARD and an alternative to biologics. It was approved by the U.S. Food and Drug Administration (FDA) on 6 November 2012 [13]. It is a Janus Kinase (JAK) inhibitor which primarily inhibits JAK1 and 3, with a reduced inhibition of JAK2 [14]. It is suggested that as a JAK1/3 inhibitor, tofacitinib might suppress cytokine/chemokine expression and the immune activation through the JAK/STAT interferon-dependent signaling pathway, thus offering a promising target for the treatment of RA [15-17].

Tofacitinib is licensed to treat adult patients with active RA in the United States, especially for those either unable to tolerate MTX or biological therapies or who have an inadequate response. During the development of tofacitinib, a series of phase II [18-21] and phase III [22-25] clinical trials were conducted in adult patients at multiple treatment centres.

To our knowledge, no systematic review has been published to evaluate the efficacy and associated AEs of tofacitinib in the treatment of RA. In this study, we undertook a systematic review with a meta-analysis of randomised controlled trials (RCTs) to investigate the efficacy and safety of tofacitinib in treating patients with RA.

Our primary objective is to compare the response rates [20% and 50% improvement in the ACR scale (ACR20) and (ACR50)] of patients receiving tofacitinib versus placebo or adalimumab. The secondary objectives are i) to compare the incidence of infections, immunological or haematological AEs in those patients receiving tofacitinib versus placebo; ii) to compare the laboratory findings in those patients receiving tofacitinib versus placebo; and iii) to compare the incidence of withdrawal from the trials in those patients receiving tofacitinib versus placebo.

## Methods

**Figure 1:**
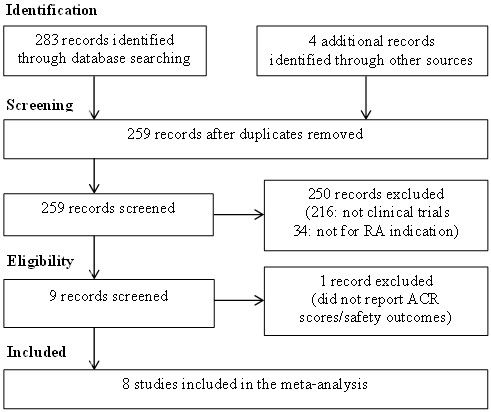
Review flowchart (PRISMA flowchart).

We performed this systematic review in accordance with the Preferred Reporting Items for Systematic Reviews and Meta-Analyses (PRISMA) Statement [26]. We searched EMBASE, the Cochrane Library and PubMed using keywords as follows: (tofacitinib) OR (tasocitinib) OR (CP-690, 550). Trial registers: the metaRegister of Controlled Trials (http://www.controlled-trials.com), the Clinical trials government (ClinicalTrials.gov) and World Health Organization International Clinical Trials Registry Platform (ICTRP) (http://www.who.int/ictrp/en/) were also searched to identify potentially relevant studies. All databases were searched on March 1, 2013. Titles, abstracts and the content of the articles were screened to determine whether the articles met the inclusion criteria. Reference lists from retrieved studies were reviewed for the identification of potentially relevant studies. The search result was presented in Figure 1.

### Inclusion criteria

The inclusion criteria for this systematic review were those published RCTs investigating the efficacy and safety of tofacitinib in adult patients (aged 18 or above) who had a diagnosis of active RA defined according to the ACR 1987 revised criteria for RA [27]. The exclusion criteria were conference proceedings as we were unable to assess the quality of these studies. Studies examining drug treatments other than tofacitinib were also excluded. Studies that did not report the primary outcomes (ACR20/50 response rates) were also excluded. Further evaluation on the full text was conducted for inclusion assessment.

### Outcome measures

The standardised response measurements for RA clinical trials of antirheumatic drugs, the ACR20 response rate and ACR50 response rate were selected as the primary outcome measures [8,28].

Secondary outcomes included the number of patients with infections, immunological or haematological AEs, such as bronchitis, influenza, nasopharyngitis, pharyngitis, rash, upper respiratory tract infection, urinary tract infection and neutropenia. Laboratory parameters examined included the least squares mean changes in neutrophil count, haemoglobin levels and serum creatinine levels, incidence of alanine aminotransferase (ALT) and aspartate aminotransferase (AST) more than one times upper limit of the normal range (ULN), mean percentage changes of low-density lipoprotein (LDL) and high-density lipoprotein (HDL). The incidence of patient withdrawal from treatment was also a secondary outcome of interest.

### Data extraction

We performed the initial electronic database searches and screened the published abstracts for eligibility. Full texts were retrieved for potentially useful articles and then screened for relevance. Those relevant articles were then assessed independently by two reviewers for inclusion in the meta-analysis.

The data relating to the primary and secondary outcomes in all included studies were extracted by two independent reviewers and cross-checked by an additional reviewer for data accuracy. Non-statistical data extracted from the eligible studies included author, study setting, study duration, doses of tofacitinib, possible concomitant medication, sample size, mean scores on 3-variable Disease Activity Score in 28 joints (DAS28-3) using C-reactive protein, mean scores on 4-variables (DAS28-4) using the erythrocyte sedimentation rate, mean scores on the Health Assessment Questionnaire disability index (HAQ-DI), and mean number of swollen joints and tender joints. Statistical data on ACR20 and ACR50 response rates, AEs and number of patient withdrawals were also extracted.

### Methodological quality assessment

The risk of bias of the identified RCT articles was assessed using the Cochrane Collaboration’s tool [29] (Additional file 1: Table S1). Assessment was conducted independently and cross-checked by additional reviewers with discrepancies resolved by consensus.

### Statistical analysis

Risk ratios (RR) and mean differences were calculated for dichotomous and continuous outcomes respectively (e.g. ACR response rates, AEs and laboratory findings). The DerSimonian and Laird random-effects model was used to deal with possible heterogeneity between studies [30]. I^2^ statistic was used to describe the proportion of the variability that is due to heterogeneity rather than sampling error. Analyses were based upon intention-to-treat (ITT) or completer analysis when ITT data was not available. When standard deviations of the outcomes were not given, they were calculated using standard errors and sample sizes. We were unable to assess publication bias by funnel plot due to the scant number of included studies. All statistical analyses were conducted using Review Manager 5.2 (Copenhagen: The Nordic Cochrane Centre, The Cochrane Collaboration, 2012). Apart from meta-analysis, the dose–response relationship was also plotted to determine the efficacy of tofacitinib at different doses at week 12. All studies were described as international multicenter trials except Tanaka *et al*.’s study [18] which was conducted solely in Japan. Therefore, sensitivity analysis was conducted by removing Tanaka *et al.*’s study [18] to examine the possible effects of differences in ethnicity.

## Results

### Search results and study selection

Figure 1 summarises the review flowchart in accordance with PRISMA statement [26]. The electronic search of EMBASE, the Cochrane Library and PubMed yielded a total of 283 studies. Four additional unpublished records were identified in the ClinicalTrials.gov, each with a different ClinicalTrial.gov identifier from the published reports (Additional file 2: Table S2). A total of 259 records were screened after the removal of the duplicates. After scanning the titles and abstracts, 216 records were removed as they were not clinical trials of tofacitinib. Full texts of 43 studies were retrieved for more detailed evaluation, of which 34 were then excluded since they were not related to the treatment of RA. Another study was excluded as it investigated pain, physical functioning and health status but not efficacy and safety measures of tofacitinib in the treatment of RA [31]. As a result, eight eligible studies were included in this systematic review, contributing a total sample size of 3,791. A standardised summary table of the included studies is presented in Table 1.

**Table 1:** Characteristics of randomised controlled studies included in this meta-analysis

**Article**	**Region**	**Study duration, weeks**	**Possible concomitant medication**	**Dose of tofacitinib**	**No of patients randomised**^**a**^	**Mean DAS28-3(CRP)**^**a**^	**Mean DAS28-4(ESR)**^**a**^	**Mean HAQ-DI**^**a**^	**Mean no of swollen joints**^**a**^	**Mean no of tender joints**^**a**^
Fleischmann 2012a [21]	United States; Europe; Latin American; the Republic of Korea	24	NSAIDs; antimalarial agents; opioids; acetaminophen; oral glucocorticoids	1, 3, 5, 10, 15 mg bid	Total: 386	Placebo:5.6	Placebo:6.6	Placebo:1.54	Placebo:16.9	Placebo:25.9
					Placebo: 59					
					tofacitinib: 54, 51, 49, 61, 57	tofacinitib: 5.5, 5.4, 5.6, 5.5, 5.5	tofacinitib: 6.5, 6.4, 6.6, 6.5, 6.5	tofacinitib: 1.57, 1.53, 1.40, 1.49, 1.62	tofacinitib: 16.7, 15.9, 17.4, 16.3, 16.9	tofacinitib: 27.0, 24.6, 27.1, 25.7, 25.9
					adalimumab 40 mg qow: 53	adalimumab 40 mg qow: 5.4	adalimumab 40 mg qow: 6.3	adalimumab 40 mg qow: 1.44	adalimumab 40 mg qow: 14.9	adalimumab 40 mg qow: 24.1
					Randomised but not treated: 2					
Fleischmann 2012b [25]	United States; Europe; Latin America; Asia	24	NSAIDs; glucocorticoids;	5, 10 mg bid	Total: 611	Placebo: 5.56	Placebo: 6.65	Placebo: 1.53	Placebo: 17.3	Placebo: 28.9
					Placebo^b^: 61, 61	tofacitinib: 5.68, 5.60	tofacitinib: 6.71, 6.70	tofacitinib: 1.53, 1.50	tofacitinib: 16.3, 17.0	tofacitinib: 29.4, 29.1
					tofacitinib:243, 245					
					Randomised but not treated: 1					
Kremer 2009 [20]	United States; Canada; Europe; Latin American	6 (treatment) + 6 (follow-up)	NSAIDs; selective COX-2 inhibitors; opioids; acetaminophen; oral glucocorticoids	5, 15, 30 mg bid	Total: 264	Placebo: 6.0	NA	Placebo: 1.7	Placebo: 20.01	Placebo: 30.3
					Placebo: 65					
					tofacitinib: 61, 69, 69	tofacitinib: 6.2, 5.7, 5.9		tofacitinib: 1.7, 1.6, 1.6	tofacitinib: 21.1, 16.2, 19.5	tofacitinib: 32.3, 26.7, 29.3
Kremer 2012 [19]	United States; Europe; Latin America	24	MTX (compulsory)	1, 3, 5, 10, 15 mg bid, 20 mg qd	Total: 509	Placebo: 5.3	Placebo: 6.1	Placebo: 1.20	Placebo: 15.7	Placebo: 21.6
					Placebo: 69	tofacitinib: 5.5, 5.3, 5.1, 5.3, 5.4	tofacitinib: 6.4, 6.1, 6.1, 6.4, 6.2	tofacitinib: 1.58, 1.36, 1.44, 1.33, 1.41	tofacitinib: 6.5, 15.7, 14.1, 14.7, 15.3	tofacitinib: 23.6, 22.8, 21.5, 24.8, 23.7
					tofacitinib: 70, 68, 71, 74, 75	20 mg qd: 5.3	20 mg qd: 6.3	20 mg qd: 1.46	20 mg qd: 15.2	0 mg qd: 23.1
					20 mg qd: 80Randomised but not treated: 2					
Tanaka 2011 [18]	Japan	12	MTX supplemented with folic acid (compulsory);	1, 3, 5, 10 mg bid	Total: 140	Placebo: 4.9	Placebo: 5.9	Placebo: 1.3	Placebo: 13.8	Placebo: 16.4
					Placebo: 28					
					tofacitinib: 28, 27, 27, 26	tofacitinib: 5.0, 5.1, 5.0, 4.9	tofacitinib: 6.1, 6.1, 6.0, 5.9	tofacitinib: 1.1, 1.3, 1.2, 1.2	tofacitinib: 13.2, 15.1, 15.6, 15.1	tofacitinib: 16.4, 16.2, 17.8, 15.4
			NSAIDs;selective COX-2 inhibitors; glucocorticoids		Randomised but not treated: 4					
van Vollenhoven2012 [22]	North America; Latin America; Europe; etc.	52	MTX (compulsory) 5, 10 mg bid		Total: 717	Placebo^c^: 5.6, 5.3	Placebo^c^: 6.6, 6.3	Placebo^c^: 1.5, 1.4	Placebo^c^: 16.9, 16.4	Placebo^c^: 26.6, 28.1
					Placebo^c^: 56, 52	tofacitinib: 5.4, 5.4	tofacitinib: 6.6, 6.5	tofacitinib: 1.5, 1.5	tofacitinib: 16.7, 15.8	tofacitinib: 28.5, 26.1
					tofacitinib: 204, 201	adalimumab 40 mg qow: 5.3	adalimumab 40 mg qow: 6.4	adalimumab 40 mg qow: 1.5	adalimumab 40 mg qow:16.4	adalimumab 40 mg qow: 26.7
					adalimumab 40 mg qow: 204					
Burmester 2013 [24]	North America, Europe, Latin America, etc.	24	MTX (compulsory); antimalarial therapy; No other DMARDs (non-biological or biological) were permitted NSAIDs, selective cyclooxygenase-2 inhibitors, or glucocorticoids	5, 10 mg bid	Total: 399	Placebo: 5.4	Placebo: 6.4	Placebo: 1.6	Placebo: 17.2	Placebo: 28.2
					Placebo^b^: 66, 66	tofacitinib: 5.4, 5.3	tofacitinib: 6.5, 6.4	tofacitinib: 1.6, 1.5	tofacitinib: 16.2, 16.6	tofacitinib: 28.4, 27.6
					tofacitinib: 133, 134					
Van der Heijde 2013 [23]	America, Europe, Asia, Australia	104	MTX (compulsory); NSAIDs; corticosteroids	5, 10 mg bid	Total: 797	Placebo^c^: 5.14, 5.18	Placebo^c^: 6.25, 6.29	Placebo^c^: 1.4, 1.23	Placebo^c^: 14.0, 14.5	Placebo^c^: 23.3, 22.6
					Placebo^c^: 81, 79	tofacitinib: 5.22, 5.20	tofacitinib: 6.34, 6.25	tofacitinib: 1.41, 1.39	tofacitinib: 14.1, 14.4	tofacitinib: 24.1, 23.0
					tofacitinib: 321, 316					

DAS28: Disease Activity Score for 28 joint counts; CRP: C-reactive protein; ESR: erythrocyte sedimentation rate; HAQ-DI: Health Assessment Questionnaire disability index; bid, twice daily; qd, once daily; qow, once every other week; NSAIDs: non-steroidal anti-inflammatory drugs; MTX: methotrexate; COX-2: cyclooxygenase-2.

^a^No. of patients in tofacitinib group are presented in ascending order of dose.

^b^Patients in placebo group were assigned to 5 or 10 mg tofacitinib after 3 months; Numbers are presented in ascending order of dose of tofacitinib after switch.

^c^Patients assigned to placebo were randomly switched to either 5 or 10 mg of tofacitinib if they did not reduce the number of swollen and tender joints by 20% after 3 months. All patients in the placebo group were assigned in a blinded fashion to either 5 or 10 mg of tofacitinib after 6 months; Numbers are presented in ascending order of dose of tofacitinib after switch.

### Methodological quality

All studies stated that they were double-blind, however half of the studies did not report the method of allocation sequence and concealment. Co-interventions and baseline characteristics were similar for the tofacitinib and placebo groups for all studies. All studies had potential risks of bias as some of the outcomes stated in the trial protocol were not reported. Another potential bias might be introduced by switching from placebo to active medication in some patients previously receiving placebo in four studies. This potential bias was addressed in two studies using the method of imputation of no response, with “advancement penalty” [22,23].

### Efficacy

**Figure 2:**
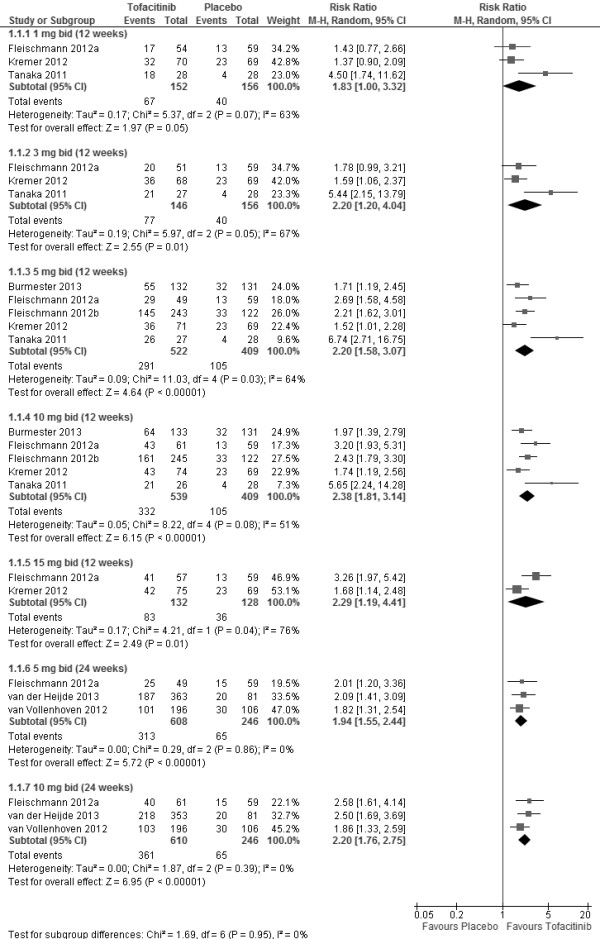
ACR20 response rates for different doses of tofacitinib at week 12 and week 24.

**Figure 3:**
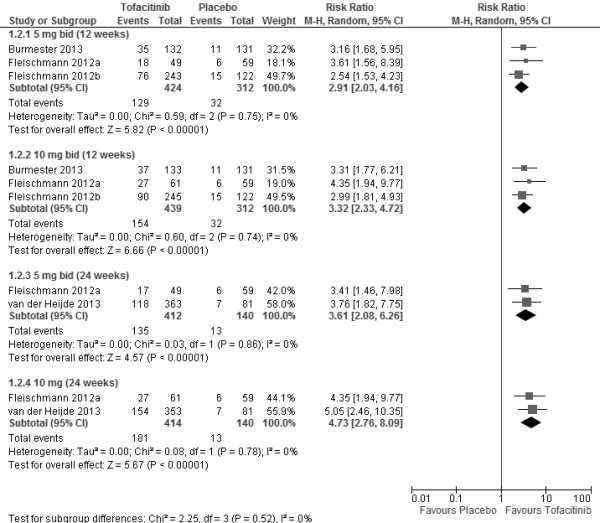
ACR50 response rates for 5 mg bid and 10 mg bid of tofacitinib at week 12 and week 24.

The doses used and the treatment duration are shown in Figures 2 and 3. ACR20 response rates were found to be significantly higher in those patients receiving tofacitinib versus placebo at doses ≥3 mg bid. A non-significant dose-dependent response trend was observed from 1 to 5 mg bid (Additional file 3: Figure S1). ACR20 response rates were significantly greater in tofacitinib treatment versus placebo at 5 mg bid (RR 2.20; 95% CI 1.58, 3.07) and 10 mg bid (RR 2.38; 95% CI 1.81, 3.14) after 12 weeks of treatment. The higher ACR20 response rates in patients receiving tofacitinib sustained at week 24 for 5 mg bid (RR 1.94; 95% CI 1.55, 2.44) and 10 mg bid (RR 2.20; 95% CI 1.76, 2.75) (Figure 2). Moreover, tofacitinib was also significantly more efficacious than placebo as measured by ACR50 response rate (Figure 3). ACR50 response rates were significantly greater in tofacitinib treatment at 5 mg bid (RR 2.91; 95% CI 2.03, 4.16) and 10 mg bid (RR 3.32; 95% CI 2.33, 4.72) after 12 weeks.

For the efficacy measures which were only reported in respective single studies, significantly higher ACR20 and ACR50 response rates were observed in patients receiving doses ≥5 mg tofacitinib versus placebo at week 6, 12 and 24 (Additional file 4: Figure S2). A significantly higher response rate was also observed in ACR50 for 3 mg tofacitinib versus placebo at week 24.

Fleischmann *et al.*[21] and van Vollenhoven *et al.*[22] also compared the efficacy of tofacitinib with adalimumab at month 3 and 6 respectively (Table 2). At month 3, there was a statistically significant difference in ACR20 response rate in patients receiving ≥5 mg bid tofacitinib versus adalimumab. At the dose of 5 mg bid, the RR of ACR20 and ACR50 response rates were 1.65 (95% CI 1.08, 2.53) and 1.95 (95% CI 1.00, 3.80) in patients receiving tofacitinib versus adalimumab respectively. The corresponding figures at 10 mg bid were 1.97 (95% CI 1.32, 2.92) and 2.35 (95% CI 1.26, 4.38) respectively. At month 6, there were no significant differences in ACR20 response rates in patients receiving tofacitinib versus adalimumab.

**Table 2:** **Risk ratios of ACR20/50 response rates of tofacitinib vs.40 mg adalimumab at month 3**[21]**and 6**[22]

	**ACR20**		**ACR50**	
**Dose of tofacitinib**	**Sample size (tofacitinib, adalimumab)**	**Risk ratio [95% CI]**	**Sample size (tofacitinib, adalimumab)**	**Risk ratio [95% CI]**
**Month 3**				
1 mg bid	54, 53	0.88 [0.52, 1.50]	54, 53	0.59 [0.23, 1.51]
3 mg bid	51, 53	1.09 [0.67, 1.80]	51, 53	1.25 [0.59, 2.63]
5 mg bid	49, 53	1.65 [1.08, 2.53]	49, 53	1.95 [1.00, 3.80]
10 mg bid	61, 53	1.97 [1.32, 2.92]	61, 53	2.35 [1.26, 4.38]
15 mg bid	57, 53	2.01 [1.35, 2.98]	57, 53	2.70 [1.46, 4.98]
**Month 6,**				
5 mg bid	196, 199	1.09 [0.89, 1.33]	N/A	N/A
10 mg bid	196: 199	1.11 [0.91, 1.36]	N/A	N/A

*bid* twice daily; *CI* confidence interval; N/A, not applicable.

### Safety

The most commonly reported infections and immune-related AEs during the 12-week tofacitinib treatment period are shown in Table 3. There were no statistically significant differences in patients receiving tofacitinib versus placebo in the incidences of infections, neutropenia and withdrawal due to AEs. However, significantly fewer patients withdrew from tofacitinib than placebo (RR 0.60; 95% CI 0.45, 0.78). Similarly, the patient withdrawal rate due to lack of efficacy was significantly lower in the patients receiving tofacitinib versus placebo (RR 0.18; 95% CI 0.09, 0.35).

**Table 3:** Adverse events with tofacitinib at week 12 and withdrawal from trials

	**5 mg bid**			**10 mg bid**		
**Adverse events**	**No. of studies**	**Sample size (tofacitinib, placebo)**	**Risk ratio [95% CI]**	**No. of studies**	**Sample size (tofacitinib, placebo)**	**Risk ratio [95% CI]**
**Infection**						
Upper respiratory tract infection	4	901, 522	1.12 [0.61, 2.05]	4	896, 522	0.77 [0.40, 1.49]
Urinary tract infection	4	901, 522	1.22 [0.58, 2.57]	4	901, 522	1.01 [0.45, 2.27]
Bronchitis	4	901, 522	0.80 [0.25, 2.56]	4	896, 522	1.01 [0.34, 2.96]
Nasopharyngitis	5	928, 550	1.57 [0.51, 4.83]	5	922,550	1.67 [0.82, 3.39]
Influenza	3	768, 390	0.37 [0.08, 1.64]	3	762, 390	1.25 [0.25, 6.20]
Pharyngitis	2	231, 136	0.01 [−0.01, 0.03]	2	227, 136	0.03 [−0.09, 0.14]
**Immune system**						
Neutropenia^a^	4	826, 482	1.41 [0.55, 3.61]	4	830, 482	1.73 [0.68, 4.38]
Rash	3	552, 296	0.32 [0.04, 2.61]	3	543, 296	2.51 [0.63, 9.93]
**Withdrawal**	**No. of studies**	**Tofacitinib N (%)**	**Placebo N (%)**			**Risk ratio [95% CI]**
All causes	5	158 (12.31%)	59 (16.71%)			0.60 [0.45, 0.78]
Adverse events	5	50 (3.89%)	8 (2.27%)			1.43 [0.68, 3.03]
Lack of efficacy	5	16 (1.25%)	20 (5.67%)			0.18 [0.09, 0.35]

*bid* twice daily; *CI* confidence interval; ^a^mild, 1500–1999 cells/mm^3^.

The mean neutrophil count significantly declined in patients receiving tofacitinib versus placebo. The mean serum creatinine was found to be significantly higher for tofacitinib 10 mg bid versus placebo. The mean percentage change of HDL/LDL was significant higher in patients receiving tofacitinib versus placebo. The RRs of the mean changes of ALT > 1 ULN and AST > 1 ULN were statistically significant (Additional file 5: Table S3).

### Sensitivity analysis

The RR of ACR20 response rate did not significantly change with the exclusion of data from Tanaka *et al.*[18]. For the tofacitinib treatment at 3 mg bid, the RR slightly reduced from 2.20 (95% CI 1.20, 4.04) to 1.65 (95% CI 1.18, 2.30), however, the heterogeneity was significantly reduced to 0% (Additional file 6: Table S4). Similarly, the RR of ACR20 response rate for the tofacitinib treatment at 5 mg bid (2.20; 95% CI 1.58, 3.07) did not change significantly (1.94; 95% CI 1.55, 2.43) but led to a reduction in heterogeneity. Although the inclusion of Tanaka *et al.*[18] led to substantial heterogeneity, it did not materially alter the conclusion. No data for ACR50 response rate was available for sensitivity analysis.

## Discussion

We undertook a rigorous systematic review and meta-analysis using independent reviewers for data extraction and statistical analysis. To our knowledge, this is the first systematic review of the RCTs investigating tofacitinib in the treatment of RA and included all relevant published RCTs to date. The meta-analysis of the clinical trials of tofacitinib in adult patients with RA showed that the use of tofacitinib 5 mg bid resulted in statistically significant higher ACR20 and ACR50 response rates compared with placebo. A non-statistically significant dose-dependent effect was observed in tofacitinib treatment at 1, 3, 5 mg bid while the effect appeared to be saturated when the dose reached 5 mg bid. In consistent with our result, the FDA has approved the clinical dose of 5 mg bid for the treatment of adults with moderately to severely active RA [32].

Apart from the comparison of efficacy in different doses, our study also compared the efficacy of tofacitinib with one of the biologics, adalimumab. However, similar to many newly marketed drugs, most of the published studies were placebo-controlled trials. Two clinical trials comparing the efficacy of tofacitinib with adalimumab were identified. Our findings showed that ACR20 and ACR50 response rates were statistically significant higher in patients receiving tofacitinib versus adalimumab at month 3, except the ACR50 response rate of tofacitinib 5 mg bid treatment. However, the ACR20 response rate of tofacitinib was similar to that of adalimumab in a period of 6 months. Aaltonen *et al.*[33] recently conducted a meta-analysis to compare the response rates (ACR20 and ACR50) of TNF inhibitors with placebo group. They reported the RR of ACR20 response rate at month 3 were 2.24 (95% CI 1.63, 3.08) and 2.50 (95% CI 1.90, 3.30) at month 6. Asltonen *et al.*’s results are also comparable to our results on tofacitinib 5 mg bid treatment at month 3 (RR 2.44; 95% CI 1.58, 3.77) and at month 6 (RR 1.87; 95% CI 1.42, 2.48). Similarly, RR of ACR50 response rate at month 3 reported by Aaltonen *et al.* was 4.16 (95% CI 2.44, 7.09) which is also comparable to ours in tofacitinib (5 or 10 mg bid) at month 3 (RR 3.05; 95% CI 2.25, 4.14). The current available evidence seemed to support the efficacy of tofacitinib in the short-term treatment of RA, which may be comparable to TNF inhibitors. However, further head-to-head direct comparison studies are needed to confirm the results.

Unlike the biologics which are administered by injection, tofacitinib is a small molecule which can be administered orally. Although tofacitinib is not currently licensed for children, an oral treatment is likely to be well received by children with MTX-resistant RA. In accordance with the requirements of the new European and FDA paediatric regulations, the manufacturer should plan on conducting paediatric clinical trials so that data will be available in the future to guide the use of tofacitinib in children.

In our meta-analysis, the results showed no statistically significant difference in the outcome of AEs in the tofacitinib group versus placebo but some laboratory abnormalities were observed in short-term studies. We found significantly higher mean serum creatinine in the tofacitinib group and it was also in line with a review reporting higher incidence rate of blood creatinine elevation in tofacitinib treatment group compared to comparator group [34]. However, this did not result in patient withdrawal at week 12 shown in our meta-analysis. Similarly, there was a significantly higher risk of ALT/AST > 1 ULN in the tofacitinib group versus placebo. One study reported that four patients discontinued in tofacitinib treatment group but none in the placebo group due to increases of AST and ALT levels [18]. Moreover, tofacitinib has the potential to cause immunosuppression, inducing serious infections and malignancies. This possibility is supported by its pharmacological action which acts as a JAK1/3 inhibitor. A conference abstract showed a statistically significant higher risk of infection due to the decrease in absolute lymphocyte counts [35]. Significantly lower neutrophil counts were also found in tofacitinib treatment versus placebo, but no patients’ discontinuation was reported. The most common infection-related AEs reported in tofacitinib treatment were upper respiratory tract infections, urinary tract infections and nasopharyngitis [27]. Tuberculosis was also reported in four patients receiving tofacitinib 10 mg bid treatment [22,23,25]. With respect to malignancies, lung cancer [22,23,25] and renal cell carcinoma [21] were descriptively reported in the included studies. Another conference abstract summarising RCTs and long-term extension studies reported higher incidence rate when the duration of exposure to tofacitinib is longer. It also reported statistically significant higher risk of lung cancer in the tofacitinib group compared with the Surveillance Epidemiology and End Result database covering the general population [36]. However more studies are needed to confirm the above findings and also investigate the risk of other malignancy associated with long-term tofacitinib treatment. Apart from malignancies, the European Medicines Agency also suggested that cardiovascular problems (largely related to adverse lipid profile) should be specifically monitored in patients with RA in clinical trials [8]. A meta-analysis reported statistically significant higher risk of hypercholesterolaemia (RR 1.70; 95%CI 1.10, 2.63) in tofacitinib treatment group when compared to the comparator group [34]. A significant increase in LDL/HDL was also noted in our meta-analysis. Of note, in one study [24], the results for changes in LDL/HDL reported in the main text was not consistent with the supplementary figures, hence was excluded from the analysis. Cardiovascular outcomes such as congestive cardiac failure, chest pain and chest discomfort were also descriptively reported in the included studies [19,21].

Despite the laboratory abnormalities, the clinical significance was still unclear as too few patients discontinued and no statistically significant difference was observed in all RRs of AEs. However, long-term pharmacovigilance studies are needed to explore the clinical significance on the laboratory abnormalities and confirm the long-term safety.

Our results showed that more patients withdrew from the placebo group than from the tofacitinib group (Table 3). The above results can be explained by the fact that significantly more patients in the placebo group withdrew due to lack of efficacy. Although there was a higher withdrawal rate due to AEs in the tofacitinib group than that of placebo group, the difference was not statistically significant. The above results further support that tofacitinib has a favorable risk/benefit ratio for short-term use.

There are several potential limitations in our meta-analysis. Firstly, all published studies have reported statistically significant higher ACR20 and ACR50 response rates in patients receiving tofacitinib when compared to placebo and all these clinical trials were sponsored by the manufacturer. At the time of the analysis, we also identified four more completed registered trials from the ClinicalTrials.gov since 2010 but peer-reviewed published results were yet to be available (Additional file 2: Table S2). Consequently, the possibility of publication bias and time-lag bias cannot be excluded. Although we have generated funnel plots to access publication bias, the paucity of the literature makes it difficult to warrant its reliability. Secondly, the patient in the included studies all had an inadequate response to MTX treatment, which may limit the generalisability of study findings to DMARD naïve patients. Thirdly, there is substantial statistical heterogeneity between studies in the outcomes of ACR20 and ACR50 response rates. This apparent heterogeneity was likely to be attributed to data from a single study by the Tanaka *et al.*[18]. However, sensitivity analysis showed that its exclusion resulted in reduction in heterogeneity without materially affecting the overall conclusions. The setting of this study was similar to all other studies in terms of concomitant medication and study duration. However we observed a high RR of ACR20 response rate, which implied that the heterogeneity may be due to the difference in study populations as this study was conducted among the Japanese population only. The other studies were conducted internationally, mainly in North America. Pharmacogenomics studies are recommended to investigate the apparent differences in efficacy.

In addition, some important information was not reported in the included studies, which limit our further understanding of the efficacy and safety of tofacitinib treatment in some circumstances. First, data according to different age groups should be reported. The manufacturer reported that elderly people (≥65 years) receiving tofacitinib might have a higher risk of developing serious infections and more severe RA symptoms, which may render different efficacy and safety of tofacitinib [27]. However, there was a lack of published information reporting the outcomes of this specific age group. Second, radiographic outcomes such as erosions, joint space narrowing and Sharp van der Heijde should be reported at least at baseline, during and at the end of the trial for assessing the efficacy [10] but they were not reported in the included trials.

## Conclusions

In conclusion, tofacitinib is more effective than placebo in the treatment of MTA-resistant RA up to 24 weeks. Tofacitinib is well tolerated as no statistically significant AEs impacting the immune or hematologic system were observed in short-term studies compared with placebo. Despite significantly lower neutrophil counts in tofacitinib group, there were no associated treatment withdrawals. However, further studies on long-term efficacy and pharmacovigilance studies are still needed to support long-term use.

## Abbreviations

ACR: American College of Rheumatology Scale; AE: Adverse event; AST: Aspartate aminotransferase; ALT: Alanine aminotransferase; BID: Twice daily; CI: Confidence interval; DAS: Disease activity score; DMARD: Disease-modifying antirheumatic drug; FDA: US food and drug administration; HAQ-DI: Health assessment questionnaire disability index; HDL: High-density lipoprotein; ICTRP: International clinical trials registry platform; ITT: Intention-to-treat; JAK: Janus Kinase; LDL: Low-density lipoprotein; MTX: Methotrexate; NICE: National institute for health and clinical excellence; NSAIDs: Non-steroidal anti-inflammatory drugs; PRISMA: Preferred reporting items for systematic reviews and meta-analyses; RA: Rheumatoid arthritis; RCT: Randomised controlled trials; RR: Relative risk; TNF: Tumor necrosis factor; ULN: Upper limit of normal.

## Competing interests

The authors declare that they have no competing interests.

## Authors’ contributions

ICKW, EWC, YH, AYSW and CSLC planned the systematic review and developed the methodology. CSLC, WCYL, KKCM, YH, AYSW, and AJW contributed to extract and interpreted the data. WCYL, KKCM, AYSW and AJW involved in the statistical analysis. YH, AYSW, WCYL, EWC and ICKW drafted the article. All authors revised the article critically for important intellectual content and approved the final manuscript.

## Pre-publication history

The pre-publication history for this paper can be accessed here:

http://www.biomedcentral.com/1471-2474/14/298/prepub

## Supplementary Material

Additional file 1: Table S1 Assessment of risk of bias in accordance to Cochrane Collaboration’s tool. Click here for file

Additional file 2: Table S2 Completed clinical trials identified from the ClinicalTrials.gov (not included in this systematic review as results were not available). Click here for file

Additional file 3: Figure S1 Risk ratios of ACR20 response rates of tofacitinib versus placebo at week 12. Click here for file

Additional file 4: Figure S2 ACR20 and ACR 50 response rates reported in three independent studies*. *The three independent studies were Kremer 2009 [20], Kremer 2012 [19] and Fleischmann 2012a [21]. Click here for file

Additional file 5: Table S3 Laboratory findings with tofacitinib treatment at week 12. Click here for file

Additional file 6: Table S4 Sensitivity analysis (exclusion of Tanaka *et al*[18]) of risk ratios of ACR20 response rates. Click here for file
