# Supplementary material for: Efficacy and safety of tofacitinib in the treatment of rheumatoid arthritis: a systematic review and meta-analysis
Source: BMC Musculoskelet Disord. 2013 Oct 18;14:298. doi: 10.1186/1471-2474-14-298 (PMC3819708; doi:10.1186/1471-2474-14-298)
Supplement: Additional file 1: Table S1 — Assessment of risk of bias in accordance to Cochrane Collaboration’s tool. [file 1471-2474-14-298-S1.doc]

Additional file 1: Table S1**. Assessment of risk of bias in accordance to Cochrane Collaboration’s tool**

| Study | Sequence generation | Allocation concealment | Blinding | Incomplete outcome data | Selective outcome reporting | Other sources of bias |
| --- | --- | --- | --- | --- | --- | --- |
| Fleischmann 2012a | Unclear | Unclear | Yes | Yes | Unclear | Yes |
| Fleischmann 2012b | Yes | Yes | Yes | Yes | Unclear | Unclear |
| Kremer 2009 | Unclear | Unclear | Yes | Yes | Unclear | Yes |
| Kremer 2012 | Unclear | Unclear | Yes | Yes | Unclear | Yes |
| Tanaka 2011 | Unclear | Unclear | Yes | Yes | Unclear | Yes |
| Van Vollenhoven 2012 | Yes | Yes | Yes | Yes | Unclear | Unclear |
| Burmester 2013 | Yes | Yes | Yes | Yes | Unclear | Unclear |
| Van der Heijde 2013 | Yes | Yes | Yes | Yes | Unclear | Unclear |

Yes: low risk of bias, No: high risk of bias, Unclear: unclear risk of bias
