# Supplementary material for: Efficacy and safety of tofacitinib in the treatment of rheumatoid arthritis: a systematic review and meta-analysis
Source: BMC Musculoskelet Disord. 2013 Oct 18;14:298. doi: 10.1186/1471-2474-14-298 (PMC3819708; doi:10.1186/1471-2474-14-298)
Supplement: Additional file 2: Table S2 — Completed clinical trials identified from the ClinicalTrials.gov (not included in this systematic review as results were not available). [file 1471-2474-14-298-S2.docx]

Additional file 2: Table S2 **Completed clinical trials identified from the ClinicalTrials.gov (not included in this systematic review as results were not available)**

| Official Title | ClinicalTrials.gov Identifier | Study Completion Date | Location | Responsible Party & Study director | Study Design | Enrolment |
| --- | --- | --- | --- | --- | --- | --- |
| Phase 3, Randomized, Double Blind, Placebo Controlled Study Of The Safety And Efficacy Of 2 Doses Of CP 690,550 In Patients With Active Rheumatoid Arthritis On Background DMARDS | NCT00856544 | January 2011 | United States;  America; Europe; Asia; Latin America | Pfizer CT.gov Call Center, Pfizer | Allocation: Randomized  Endpoint Classification: Safety/Efficacy Study  Intervention Model: Parallel Assignment  Masking: Double Blind (Subject, Caregiver, Investigator, Outcomes Assessor)  Primary Purpose: Treatment | 795 |
| An Exploratory Phase 2a, Randomized, Double-Blind, Placebo-Controlled, Multicenter Study To Assess The Pharmacodynamics Of CP-690,550, Administered Orally Twice Daily (bid) For 4 Weeks, In Subjects With Active Rheumatoid Arthritis | NCT00976599 | July 2011 | United States | Pfizer CT.gov Call Center, Pfizer | Allocation: Randomized  Endpoint Classification: Pharmacodynamics Study  Intervention Model: Parallel Assignment  Masking: Double Blind (Subject, Caregiver, Investigator, Outcomes Assessor)  Primary Purpose: Basic Science | 29 |
| A Phase 2, Randomized, Double-Blind, Placebo-Controlled, Multicenter Study To Confirm Dose Responsiveness Following 12 Weeks Of The Administration Of CP-690,550 (5 Doses) Or Placebo In Subjects With Active Rheumatoid Arthritis Inadequately Responding To At Least 1 DMARD | NCT00687193 | July 2010 | Japan | Pfizer CT.gov Call Center, Pfizer | Allocation: Randomized  Endpoint Classification: Safety/Efficacy Study  Intervention Model: Parallel Assignment  Masking: Double Blind (Subject, Caregiver, Investigator, Outcomes Assessor)  Primary Purpose: Treatment | 318 |
| A Phase 2B, Randomized, Double Blind, Placebo-Controlled, Multicenter Study To Compare 6 Dose Regimens Of CP-690,550 Vs. Placebo, Each Combined With Methotrexate, Administered For 6 Months In The Treatment Of Subjects With Active Rheumatoid Arthritis Who Have Had An Inadequate Response To Methotrexate Alone | NCT00413660 | August 2008 | United States; Europe | Pfizer CT.gov Call Center Pfizer | Allocation: Randomized  Endpoint Classification: Safety/Efficacy Study  Intervention Model: Parallel Assignment  Masking: Double Blind (Subject, Caregiver, Investigator, Outcomes Assessor)  Primary Purpose: Treatment | 509 |
