# Supplementary figures and images for: Efficacy and safety of tofacitinib in the treatment of rheumatoid arthritis: a systematic review and meta-analysis
Source: BMC Musculoskelet Disord. 2013 Oct 18;14:298. doi: 10.1186/1471-2474-14-298 (PMC3819708; doi:10.1186/1471-2474-14-298)

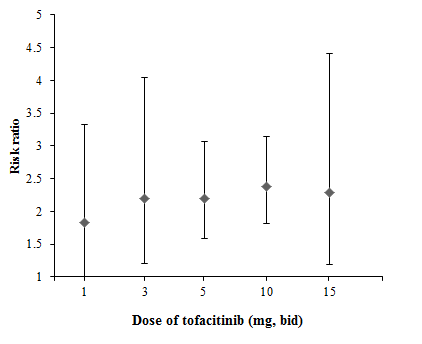

Supplement: Additional file 3: Figure S1 — Risk ratios of ACR20 response rates of tofacitinib versus placebo at week 12. [file 1471-2474-14-298-S3.tiff]

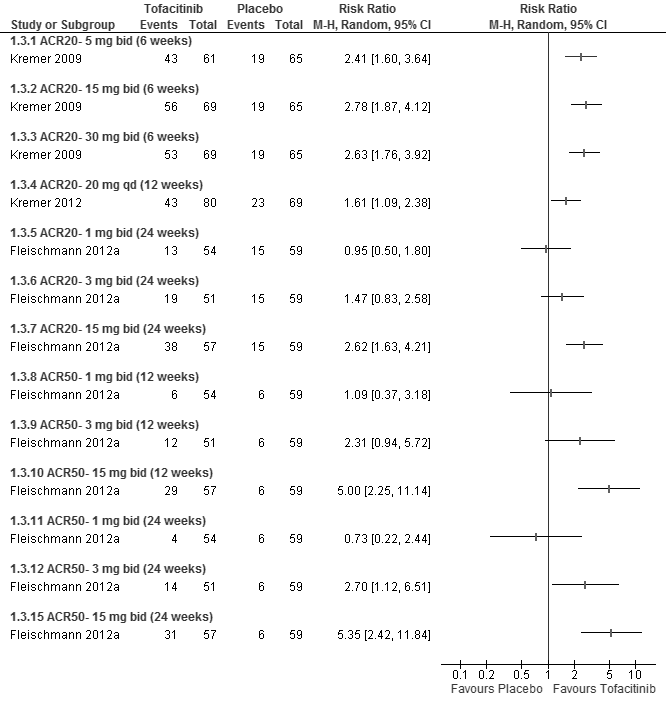

Supplement: Additional file 4: Figure S2 — ACR20 and ACR 50 response rates reported in three independent studies*. *The three independent studies were Kremer 2009 [20], Kremer 2012 [19] and Fleischmann 2012a [21]. [file 1471-2474-14-298-S4.tiff]
