# Supplementary material for: Efficacy and safety of tofacitinib in the treatment of rheumatoid arthritis: a systematic review and meta-analysis
Source: BMC Musculoskelet Disord. 2013 Oct 18;14:298. doi: 10.1186/1471-2474-14-298 (PMC3819708; doi:10.1186/1471-2474-14-298)
Supplement: Additional file 5: Table S3 — Laboratory findings with tofacitinib treatment at week 12. [file 1471-2474-14-298-S5.doc]

**Additional file 5: Table S3. Laboratory findings with tofacitinib treatment** at week 12

|  | 5 mg bid | | | 10 mg bid | | | |
| --- | --- | --- | --- | --- | --- | --- | --- |
| **Laboratory parameters** | No. of studies | Sample size  (tofacitinib, placebo) | Mean difference  [95% CI] | No. of studies | | Sample size  (tofacitinib, placebo) | Mean difference [95% CI] |
| **Mean change from baseline** |  |  |  |  | |  |  |
| Neutrophil count, 10^-3^/mm^3^ | 3 | 580, 362 | -0.75  [-1.07, -0.44] | 3 | | 580, 362 | -0.92  [-1.37, -0.46] |
| Haemoglobin, g/dl | 3 | 580, 362 | 0.14  [-0.11, 0.40] | 3 | | 580, 362 | 0.09  [-0.05, 0.23] |
| Serum creatinine, mg/dl | 3 | 580, 362 | 0.03  [-0.00, 0.06] | 3 | | 580, 362 | 0.04  [0.01, 0.07] |
| Low-density lipoprotein (%) | 2 | 447, 230 | 10.87  [6.74, 14.99] | 2 | | 446, 230 | 16.90  [12.75, 21.05] |
| High-density lipoprotein (%) | 3 | 738, 373 | 12.77  [10.14, 15.40] | 3 | | 741, 373 | 15.29  [12.65, 17.93] |
|  | 5 mg bid | | | 10 mg bid | | | |
| **Laboratory parameters** | No. of studies | Sample size  (tofacitinib, placebo) | Risk Ratio  [95% CI] | No. of studies | Sample size  (tofacitinib, placebo) | | Risk Ratio  [95% CI] |
| **Mean change from baseline** |  |  |  |  |  | |  |
| ALT>1ULN | 4 | 648, 490 | 1.81  [1.07, 3.07] | 4 | 645, 490 | | 1.99  [1.52, 2.60] |
| AST>1ULN | 4 | 620, 458 | 2.21  [1.24, 3.96] | 4 | 615,458 | | 2.26  [1.26, 4.03] |

bid, twice daily; CI, confidence interval; ALT, alanine aminotransferase; AST, aspartate aminotransferase; ULN, upper limit of the normal range
