# Supplementary material for: Efficacy and safety of tofacitinib in the treatment of rheumatoid arthritis: a systematic review and meta-analysis
Source: BMC Musculoskelet Disord. 2013 Oct 18;14:298. doi: 10.1186/1471-2474-14-298 (PMC3819708; doi:10.1186/1471-2474-14-298)
Supplement: Additional file 6: Table S4 — Sensitivity analysis (exclusion of Tanaka et al[18]) of risk ratios of ACR20 response rates. [file 1471-2474-14-298-S6.doc]

Additional file 6: Table S4. Sensitivity analysis (exclusion of Tanaka *et al* [18]) of risk ratios of ACR20 response rates

| Dose of tofacitinib | Original model | | | Exclusion of *Tanaka et al* | | |
| --- | --- | --- | --- | --- | --- | --- |
|  | Sample size | Risk ratio  [95% CI] | I^2^ | Sample size | Risk ratio  [95% CI] | I^2^ |
|  | (tofacitinib, placebo) |  |  | (tofacitinib, placebo) |  |  |
| 1 mg bid | 152, 156 | 1.83 [1.00, 3.32] | 63% | 124, 128 | 1.39 [0.98, 1.97] | 0% |
| 3 mg bid | 146, 156 | 2.20 [1.20, 4.04] | 67% | 119, 128 | 1.65 [1.18, 2.30] | 0% |
| 5 mg bid | 522, 409 | 2.20 [1.58, 3.07] | 64% | 495, 381 | 1.94 [1.55, 2.43] | 25% |
| 10 mg bid | 539, 409 | 2.38 [1.81, 3.14] | 51% | 513, 381 | 2.21 [1.77, 2.77] | 31% |

CI, confidence intervals; bid, twice daily
